# Gene flow and an anomaly zone complicate phylogenomic inference in a rapidly radiated avian family (Prunellidae)

**DOI:** 10.1186/s12915-024-01848-7

**Published:** 2024-02-27

**Authors:** Zhiyong Jiang, Wenqing Zang, Per G. P. Ericson, Gang Song, Shaoyuan Wu, Shaohong Feng, Sergei V. Drovetski, Gang Liu, Dezhi Zhang, Takema Saitoh, Per Alström, Scott V. Edwards, Fumin Lei, Yanhua Qu

**Affiliations:** 1 Key Laboratory of Zoological Systematics and Evolution, Institute of Zoology, Chinese Academy of Sciences, Beijing, China; 2 College of Life Sciences, University of Chinese Academy of Sciences, Beijing, China; 3 Department of Bioinformatics and Genetics, Swedish Museum of Natural History, PO Box 50007, Stockholm, SE-104 05 Sweden; 4 Jiangsu International Joint Center of Genomics, Jiangsu Key Laboratory of Phylogenomics & Comparative Genomics, School of Life Sciences, Jiangsu Normal University, Xuzhou, 221116 Jiangsu China; 5 Center for Evolutionary & Organismal Biology, Zhejiang University School of Medicine, Hangzhou, 310058 China; 6 National Museum of Natural History, Smithsonian Institution, Washington, DC 20004 USA; 7 Present address: U.S. Geological Survey, Eastern Ecological Science Center at Patuxent Research Refuge, Laurel, MD 20708 USA; 8 Chinese Academy of Forestry, Institute of Ecological Conservation and Restoration, Beijing, 100091 China; 9 Yamashina Institute for Ornithology, Abiko, Chiba Japan; 10 Animal Ecology, Department of Ecology and Genetics, Evolutionary Biology Centre, Uppsala University, Norbyvägen 18 D, 752 36 Uppsala, Sweden; 11 Museum of Comparative Zoology and Department of Organismic & Evolutionary Biology, Harvard University, 26 Oxford Street, Cambridge, MA 02138 USA; 12 Liangzhu Laboratory, Zhejiang University, 1369 West Wenyi Road, Hangzhou, 311121 China; 13 Innovation Center of Yangtze River Delta, Zhejiang University, Jiashan, 314102 China

**Keywords:** Speciation, Phylogenomics, Topological incongruence, Interspecific introgression, Recombination rate, Z chromosome

## Abstract

**Background:**

Resolving the phylogeny of rapidly radiating lineages presents a challenge when building the Tree of Life. An Old World avian family Prunellidae (Accentors) comprises twelve species that rapidly diversified at the Pliocene–Pleistocene boundary.

**Results:**

Here we investigate the phylogenetic relationships of all species of Prunellidae using a chromosome-level de novo assembly of *Prunella strophiata* and 36 high-coverage resequenced genomes. We use homologous alignments of thousands of exonic and intronic loci to build the coalescent and concatenated phylogenies and recover four different species trees. Topology tests show a large degree of gene tree-species tree discordance but only 40–54% of intronic gene trees and 36–75% of exonic genic trees can be explained by incomplete lineage sorting and gene tree estimation errors. Estimated branch lengths for three successive internal branches in the inferred species trees suggest the existence of an empirical anomaly zone. The most common topology recovered for species in this anomaly zone was not similar to any coalescent or concatenated inference phylogenies, suggesting presence of anomalous gene trees. However, this interpretation is complicated by the presence of gene flow because extensive introgression was detected among these species. When exploring tree topology distributions, introgression, and regional variation in recombination rate, we find that many autosomal regions contain signatures of introgression and thus may mislead phylogenetic inference. Conversely, the phylogenetic signal is concentrated to regions with low-recombination rate, such as the Z chromosome, which are also more resistant to interspecific introgression.

**Conclusions:**

Collectively, our results suggest that phylogenomic inference should consider the underlying genomic architecture to maximize the consistency of phylogenomic signal.

**Supplementary Information:**

The online version contains supplementary material available at 10.1186/s12915-024-01848-7.

## Background

Reconstructing phylogenetic relationships for rapidly radiating groups has proven to be particularly difficult [1–4]. This is because rapid radiations are particularly prone to extensive incomplete lineage sorting (ILS) and resulting high gene-tree discordance, which can result in unresolved or poorly resolved nodes in species trees [5–8]. Moreover, the close evolutionary relationships among rapidly radiated species also create opportunities for gene flow, which can lead to additional gene tree-species tree conflicts [9–11]. In cases of extreme gene tree conflicts, the most common gene tree does not match that of the underlying species tree, resulting in anomalous gene trees, a so-called “anomaly zone” [12, 13]. There is an increasing number of cases that have revealed situations where ILS can produce an anomaly zone in the species tree, partially because it is the most common form of biological topological incongruence [5]. Additionally, recent simulation studies have demonstrated that anomalous gene trees can occur in the presence of gene flow and thus produce a gene flow anomaly zone [14, 15], but whether these observations apply to empirical data is yet unknown. For example, Edwards [5] did not consider gene flow as an explanation for incongruence; given the ubiquity of gene flow in recent population genomic and phylogenomic studies [16], it could be an important driver of signals for the anomaly zone. It is, therefore, imperative for empirical studies to consider the underlying gene tree support for the inferred species trees.

Genome-wide phylogenetic inference, i.e., phylogenomics, has increased the potential for understanding how processes such as ILS and introgression can affect phylogenetic reconstruction in cases of rapid diversification [3, 17, 18]. In phylogenomics, phylogenetic reconstruction typically utilizes thousands of orthologous loci or whole-genome sequence data (e.g., [1, 18–20]). Relatively fast-evolving markers such as introns have been often found to contain more phylogenetic signal (i.e., greater support for a particular topology and less among-locus conflict) than protein-coding sequences, and have been regarded as a promising genomic resource to resolve problems caused by ILS [4, 21–23]. Additionally, it has been shown that coalescent tree approaches are often better at dealing with ILS during phylogenetic reconstruction than concatenation approaches, especially when ILS is common, because they explicitly attempt to accommodate gene tree heterogeneity [5, 24, 25].

When assuming that all discordance among genetic loci results from ILS, inference of coalescent trees does not account for topological discordance stemming from gene tree estimation errors and introgression (e.g., [26–31]). For example, gene tree heterogeneity can be caused by various forms of gene tree reconstruction errors, e.g., insufficient phylogenetic information, inadequate models of evolution, improper alignments of sequences, and inference error (e.g., [32]). In addition, interspecific gene flow is known to be an important contributor to gene tree heterogeneity. Supposition that all gene tree heterogeneity is the result of ILS may cause inaccurate species tree inference (e.g., [33]). It has been suggested that, for species with extensive hybridization, signatures of ancient branching events can be depleted from chromosomal segments in regions with high rates of recombination because introgressed deleterious alleles are more efficiently unlinked from neutral or positively selected variants [11, 34–36]. Consequently, if introgression has been pervasive in the speciation history, genomic regions of low recombination may contain phylogenetic signal that is more useful in reconstructing the putative species tree than regions with high recombination rates (e.g., [11, 36]).

Here we explore the effects of ILS, introgression, and variation in recombination rate on phylogenetic reconstruction in a group of rapidly diversifying birds, the Accentors, Prunellidae [37, 38]. The accentors are a close-knit group consisting of twelve currently recognized species [39, 40]. They are primarily distributed across the mountains of the Palearctic and vary in their elevational and habitat preferences from high alpine zones to lower montane regions and forested plains. Previous phylogenetic analyses of the accentors based on mitochondrial and up to ten nuclear loci show that these species diversified rapidly between the mid-Pliocene and early Pleistocene [37, 38, 41]. As several species diversified almost simultaneously in the early Pleistocene, this may have led to ILS and poorly resolved phylogenetic relationships. Furthermore, the distributional ranges of these primarily montane birds may have shifted during the Pleistocene glacial cycles, which may have provided opportunities for secondary contact and gene flow between the species [41]. As such, the historical gene flow may have caused gene tree conflicts and difficulties for phylogenetic reconstruction. Herein this group of birds provides a unique opportunity to investigate how ILS and introgression and the resultant anomaly zone (if any) affect phylogenomic reconstruction in the rapidly radiated groups.

To explore this, we estimated coalescent and concatenated species trees using exonic and intronic datasets obtained from a chromosome-level genome assembly of the Rufous-breasted accentor (*Prunella strophiata*), and 36 resequenced genomes generated from all twelve species of accentors. We postulate that if ILS is the major cause of gene tree conflicts, we would expect coalescent methods to recover a more congruent and well-supported topology than concatenated inferences, and we expect that ILS under the coalescent simulation could explain most of the observed gene tree heterogeneity. However, if introgression is the predominant process causing topological incongruence among gene trees, we would expect to observe signs of such introgression. Consequently, gene tree topology concentrated to regions with low recombination rate would be more resistant to interspecific introgression. We also explored whether ILS and/or introgression would produce an anomaly zone, and if so, what this would mean for the phylogenetic reconstruction. Overall, our integrative approach provides a useful framework for evaluating multiple processes underlying phylogenetic incongruence during rapid diversification events.

## Results

### De novo genome of Prunella strophiata

We first generated a de novo genome assembly from a male individual of *P. strophiata* (Voucher ID XZ15142, Linzie, Tibet) using both Illumina short-read and PacBio long-read sequencing data. PacBio library was sequenced on 17 cells using the PacBio Sequel II platform, yielding 60.35 Gb of cleaned data corresponding to ~ 57-fold coverage of the *P. strophiata* genome assembly. We constructed 500-bp library and sequenced 150-bp short reads on the Illumina NovaSeq platform. This yielded 50 Gb of cleaned data corresponding to ~ 48-fold coverage of the genome. Our genome assembly contained 2530 contigs spanning 1.055 Gb with a contig N50 of 9.754 Mb (Additional file 1: Table S1). BUSCO estimated that the assembly of *P. strophiata* contained 90% eukaryote_odb9 BUSCO orthologues (Additional file 1: Table S2). Hi-C linking information was used to further anchor, order, and orient these contigs resulting in 33 chromosomes-level scaffolds, which included 32 autosomes and the sex chromosome Z (Additional file 1: Table S3). Approximately 97% of the assembled bases were anchored to the chromosomes-level scaffolds. *P. strophiata* genome showed conserved collinearity with that of *T. guttata* (Additional file 1: Fig. S1).

### Phylogenomic relationships of accentors inferred using intron-set and exon-set

We included all currently recognized species of Prunellidae (Additional file 1: Table S4), which consists of a single genus (*Prunella*) with twelve species [40, 42]. Thirty-four re-sequenced genomes from Prunellidae, two from the tree sparrow and one from the Red-banded flowerpecker (the latter three individuals were used as outgroups) were mapped against the *P. strophiata* genome with an average 21-fold coverage (Additional file 1: Table S5). We first searched sequence homologs of intronic and exonic loci across the chromosomes using alignments from four passerine species (*Acanthisitta chloris*, *Corvus brachyrhynchos*, *Geospiza fortis*, and *Manacus vitellinus*) generated by Jarvis et al. [1]. We filtered all alignments shorter than 100 bp and have also checked the alignments manually to remove those that included non-homologous sequences for some taxa (indicated by an extreme proportion of variable positions in the alignment) and those that contained no phylogenetic information (no parsimony-informative sites). We obtained a total of 6879 intronic and 2373 exonic loci that span a length of 7,044,827 bp and 1,376,462 bp, respectively. The average sequence divergence observed among the accentor alignments was 1.97% (0.17–3.06%) for intron-set and 1.48% (0.24–3%) for exon-set, respectively.

**Fig. 1:**
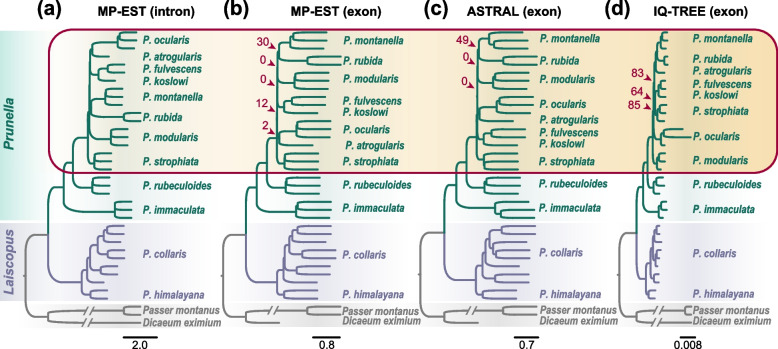
Phylogenetic estimations based on the intron-set and exon-set. **a** Coalescent and concatenated species trees inferred from the intron-set show the same topology (only the MP-EST species tree is shown here). **b**–**d** Coalescent (**b** and **c**, generated with MP-EST and ASTRAL, respectively) and concatenated phylogenies (**d**, generated with IQ-TREE) derived from the exon-set. All four trees support monophyly of the subgenera *Prunella* (shaded by light green) and *Laiscopus* (shaded by light blue). Within *Prunella*, the four phylogenetic trees also support the basal splits of *P. immaculata* and *P. rubeculoides*. However, the relationships among the remaining species differ between the concatenated and coalescent trees inferred from the exon-set and intron-set (indicated by red box). Bootstrap supports exceed 90% at all nodes except those marked by red arrows

We used both concatenated and coalescent approaches to estimate phylogenomic relationships of the accentors for the intron-set and exon-set using IQ-TREE [43], ASTRAL-III v5.6.3 [44, 45], and MP-EST v2.1 [46]. The concatenated species trees, ASTRAL and MP-EST species trees inferred from the intron-set, produced identical phylogenies (Fig. 1a). The twelve species of accentors fell into two primary clades, *Laiscopus* and *Prunella*. The *Laiscopus* clade included the *P. himalayana* and *P. collaris*, while the *Prunella* clade consisted of the remaining ten species. Within the *Prunella* clade, the *P. immaculate*, *P. rubeculoides*, *P. strophiata*, and *P. modularis* formed four successive single-species lineages, while the remaining six species were recovered as a monophyletic subclade that comprised three minor subclades. Within one of these minor subclades, the *P. atrogularis* was recovered as sister to *P. ocularis*, while a second minor subclade included *P. fulvescens* and *P. koslowi* as a sister pair. These two subclades formed a clade that in turn was sister to a third minor subclade comprising *P. rubida* and *P. montanella* (Fig. 1a).

The phylogenomic analyses of the exon-set using concatenated and coalescent approaches yielded roughly similar topologies to those of the intron-set (Fig. 1b–d). However, within the *Prunella* clade, there was a notable discordance in the positions of the some species across the three exon-set phylogenies, as well as between them and the intron-set phylogenies (Fig. 1a–d). First, *P. modularis* was the sister to the *P. montanella* and *P. rubida* pair in the MP-EST and ASTRAL species trees based on exon-set with relative weak bootstrap support (BS, 51 and 65% in ASTRAL and MP-EST species trees, respectively), but formed a single-species lineage in the intron-set-based phylogenies and concatenated exonic tree with 100% BS. Second, the *P. fulvescens* and *P. koslowi* grouped with *P. strophiata* in the exon-set based concatenated tree (although with a relative weak BS of 52%), or formed a single lineage, as in the MP-EST and ASTRAL species trees, whereas *P. fulvescens*/*P. koslowi* formed the sister clade to the *P. ocularis*/*P. atrogularis* pair in all intron-set-based phylogenies. Third, *P. atrogularis* was the sister to the *P. montanella* and *P. rubida* pair in the exon-set based concatenated tree (with 100% BS), but sister to *P. ocularis* in all intron-set-based phylogenies and exon-set-based MP-EST and ASTRAL species trees (all with 100% BS). In summary, phylogenomic analyses with intron-set and exon-set showed different topologies that are restricted to a subclade of the *Prunella* clade with a few short, internal branches subtending various pulses of diversification of a few species. As such, both the coalescent and concatenated methods cannot resolve the phylogenetic relationships of these species.

### Test topological difference between estimated gene trees and species trees

**Fig. 2:**
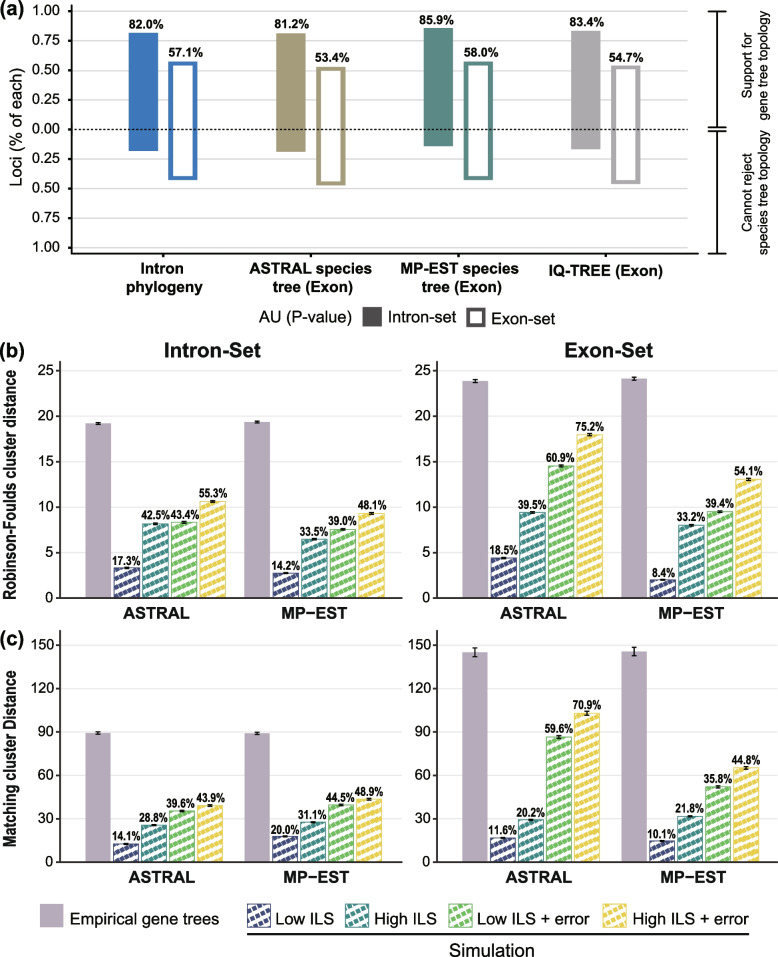
Support for observed topological discordance in the estimated gene trees. **a** Support that observed gene tree topologies differs from each of the inferred species trees, i.e., phylogeny inferred from intron-set (blue), ASTRAL species tree inferred from exon-set (olive green), MP-EST species tree inferred from exon-set (dark green), and concatenated tree inferred from exon-set (gray). Bars showed the proportion of loci that reject and fail to reject the species tree topology at a Bonferroni-corrected *P* value of 0.05 (AU tests). **b,c** Pairwise distances between gene trees and MP-EST and ASTRAL species tree. Distance between each gene tree and the species tree topology was calculated as the Robinson-Foulds cluster distance (**b**) and matching cluster distance (**c**). The mean values for all pairwise gene tree-species tree distance within each category are shown. Error bars indicate the 95% confidence interval of the mean. Distances were calculated from empirically estimated gene trees and from data sets of 1500 gene trees that were simulated using coalescent branch lengths from the MP-EST and ASTRAL species trees. Values above bars for simulated data sets indicate the ratios of means for simulated data sets compared to the mean for empirically estimated gene trees

We next investigated whether the topological differences between the estimated gene trees and the species trees were well supported. The approximately unbiased (AU) statistic tested whether the gene trees fit the species tree topology, and showed that approximately 53–58% of the exonic and 81–86% of the intronic gene trees rejected the species tree topology at a Bonferroni corrected *P* < 0.05 (Fig. 2a).

Coalescent simulations were used to assess what proportion of the total gene tree conflict was likely attributable to ILS and gene tree estimation errors. We first simulated gene trees using the coalescent branch lengths of the MP-EST and ASTRAL species trees under the assumption of low and high ILS, respectively, for both the intron-set and exon-set. As indicated from the Robinson-Foulds (RF) distance and the matching cluster distance, gene tree heterogeneity estimated from the simulated gene trees under the low and high ILS accounted for 14–20% and 29–43% of those estimated from empirical intronic gene trees, and 8–19% and 20–39% of those estimated from the empirical exonic gene trees (Fig. 2b,c). When taking gene tree estimation error into account, gene tree heterogeneity from the stimulated gene trees accounted for 39–45% and 44–55% of those empirical intronic gene trees under the low and high ILS, and 36–61% and 45–75% of empirical exonic gene trees under the low and high ILS, respectively (Fig. 2b,c). These results suggest that ILS and gene tree estimation error, even of assuming the highest extent of ILS, cannot produce the observed gene tree discordance. We thus speculated whether another process, e.g., introgression, can explain the observed topological incongruence.

### Testing for introgression

**Fig. 3:**
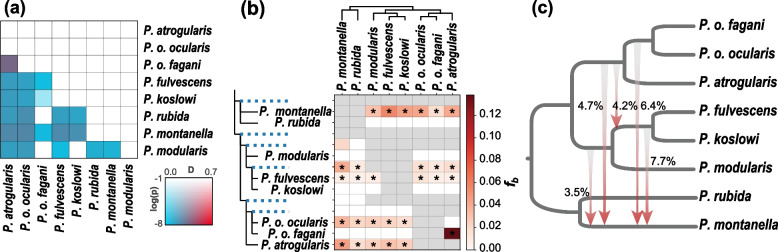
Topological incongruence and interspecific introgression. **a** Patterson’s *D* statistic values for the every trio of the seven species in the *Prunella* clade (eight taxa as *P. o. ocularis* and *P. o. fagani* were treated separately) that fall into the anomaly zone. The color legend is corresponding to the *P* value (Block-jackknife procedure to estimate *P* values) and magnitude of gene flow (*D*-statistic values). **b** Identifying possible introgression events using branch-specific statistic *f*_*b*_ (P3). The excess sharing of derived alleles between the branches of the tree is on the *y*-axis and species P3 on the *x*-axis. The ASTRAL species tree inferred from the genomic regions with low recombination rate was used as a guide tree in the analysis. Colors correspond to *f*_*b*_ values and asterisks denote block jackknifing significance at *Z* > 3 (FDR-adjusted *P* < 0.001). **c** Estimated proportions of introgression segments resulting from ancestral hybridization events

We applied three methods to detect whether introgression occurred among the seven species that are involved in the topological conflict. First, we calculated Patterson’s *D* statistics for every trio of the seven species to explore the potential gene flow between species pairs, without setting any prior topology. We detected extensive introgression for most comparisons by showing significantly non-zero *D* values (block-jackknifing significance Z > 3, FDR-adjusted *P* < 0.001, Fig. 3a). Second, based on the topology inferred from the low-recombination regions of the Z chromosome as the likely species tree (see below), we used *f*-branch metric (*f*_b_) to identify introgression to specific internal branches. This statistic reflects excess sharing of alleles between the species (identified as P3) and the descendants of the branch labeled *b* (A), relative to allele sharing between P3 and the descendants of the sister branch of *b* (B). Using a threshold of *Z* score > 3 and FDR-adjusted *P* < 0.001, we identified that 24.1% (27 out of 112 comparisons) *f*_b_ (P3) values were significantly elevated. The majority of the introgression events were between *P. montanella* and other accentors (37%), and between *P. fulvescens* and others (33%). We also observed significantly increased *f*_b_ values between *P. montanella*/*P. rubida* and the ancestor of *P. fulvescens*/*P. koslowi*, suggesting ancestral introgression (Fig. 3b).

To identify the signatures of ancient introgression, we used five-taxon comparisons performed with *D*_FOIL_ to quantify the introgressed genomic segments between interlineages, which indicate the unique signatures of ancient introgression that are consistently retained in each descendant species. We observed striking patterns of interlineage introgression (chi-square goodness-of-fit test, *P* < 0.001, Fig. 3c). For example, we identified three episodes of ancient introgression involving *P. montanella*, (1) with the ancestor of *P. atrogularis*, *P. o. ocularis* and *P. o. fagani*, (2) with the ancestor of *P. fulvescens* and *P. koslowi*, and (3) with the ancestor of *P. fulvescens*, *P. koslowi*, and *P. modularis*. Taken together, these results suggest multiple episodes of introgression among the seven species that showed great extent of topological incongruence.

### Presence of an empirical anomaly zone

**Fig. 4:**
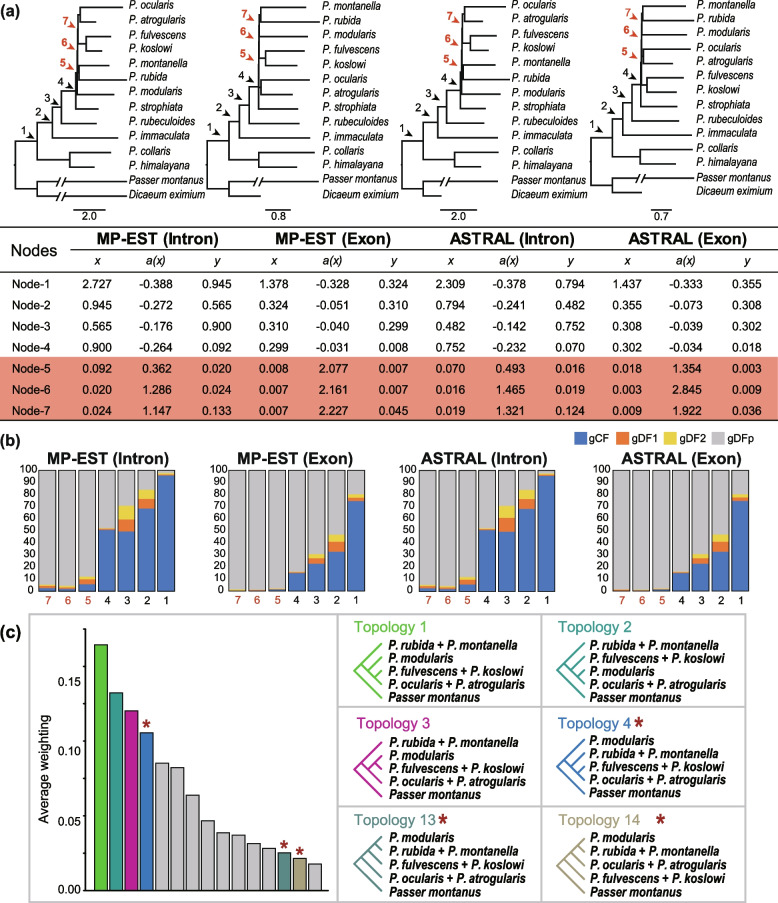
Short successive internal branches and gene tree topology distribution indicate presence of an empirical anomaly zone. **a** ASTRAL and MP-EST species tree topologies for intron-set and exon-set are shown with internal branch lengths in coalescent units. Terminal branch lengths are uninformative and are drawn as a constant value across taxa. Coalescent branch lengths for all pairs of branches (*x* and *y*) are given below, with *a*(*x*) calculated as described in Ref. [12]. Anomaly zone are expected when *y* < *a*(*x*). Clades fulfilling this anomaly zone criterion are marked (red arrows). **b** Gene concordance factors (gCFs, blue bars) for the nodes (1–7) that support the species tree (upper) and the two most common alternative topologies (gDF1 and gDF2, orange and yellow bars, respectively). The gray bars (gDFp) are the relative frequencies of all other topologies. The nodes showing lower concordance factors (5, 6, and 7) represent the lineages that fall into anomaly zone, with a remarkable number of alternative topologies. **c** Topology distribution of the four lineages consisting of the subclade of *Prunella* falling into anomaly zone. The most common topology (topology 1 indicated by green) occurring only in 16% of 50-kb windows. The topology recovered by the intron-set based phylogeny (topology 4 indicated by blue), the MP-EST species tree (topology 13 indicated by dark green) and the ASTRAL species tree (topology 14 indicated by olive green) inferred from the exon-set are the fourth (12% of 50-kb windows), thirteenth (2.5% of 50-kb windows), and fourteenth (2.1% of 50-kb windows) most commonly observed topologies (marked by red stars), respectively

For both the intron-set and exon-set, most gene trees do not support any of the candidate species trees, thereby suggesting the possibility of an empirical anomaly zone. We then followed Degnan and Rosenberg [12] to identify the potential anomaly zone. We identified that the branch lengths in coalescent units estimated with either the MP-EST or the ASTRAL species trees have values expected to produce anomaly zone across the three short successive internal branches that separate *P. modularis*, *P. montanella*/*P. rubida*, *P. fulvescens*/*P. koslowi*, and *P. ocularis*/*P. atrogularis*, as these three consecutive short internal branches lay within the limits of the anomaly zone (i.e., *y* < *a*[*x*], Fig. 4a). The position of the identified anomaly zone is same on the intron-set and exon-set phylogenies.

As Huang and Knowles [47] pointed out, gene tree discordance can also be produced by the presence of many uninformative genes. Consequently, for a species tree with short branches, in practice many gene trees underlying an inference of an anomaly zone could instead by polytomies. Given this, we performed polytomy tests across the intron-set and exon-set based ASTRAL and MP-EST coalescent species trees to test whether the three successive short internal branches represent a polytomy rather than an anomaly zone. We showed that polytomy can be rejected along these internal branches in the intron-set-based coalescent trees although they failed to reject the polytomy hypothesis in exon-set-based phylogenies (*P* < 0.05, Additional file 1: Fig. S2).

However, the approach described in Degnan and Rosenberg [12] assumes that no gene flow follows speciation, an assumption that is violated in the case of Prunellidae. As anomalous gene tree is unrooted gene tree that does not match the species tree, yet has a higher probability than the topology matching the species tree, we therefore estimated gene tree frequency on a clade-by-clade basis by calculating the quartet gCF and gDF (gene trees concordant or discordant with species tree-like topology) as described in Solís-Lemus et al. [14] and Long and Kubatko [15]. The gDFs of the two quartets that disagree with the “major” quartet displayed by the species tree (i.e., gDF1 and gDF2) are greater than the gCF of the major quartet, creating an anomaly zone. Consistent with this, we found that gDF1 or gDF2 occurred at roughly similar to (i.e., introns) or higher (i.e., exons) than the major quartet (gCF) at the three nodes (i.e., nodes 5–7), supporting the existence of anomalous gene tree in this part of species tree (Additional file 1: Fig. Table S6). Conversely, for the remaining four nodes (i.e., nodes 1–4), gCF values were higher than those of the two gDF values, suggesting that major quartets displayed by this part of species tree are typically supported by the majority of individual gene trees.

As gCF and gDF statistics were estimated by using loci from parts of chromosomes, i.e., exons and introns, we further tested the existence of anomalous gene trees in the nodes 5–7 by using whole chromosome data. Since this anomaly zone includes four simultaneously diversifying lineages, we investigated the topology distribution of these four lineages in 50-kb non-overlapping sliding windows using topology weight analysis (TWISST) [48]. We found that the most common topology occurred in 16% of the windows, which was not recovered by any coalescent or concatenated inference phylogenies (Fig. 4c). Conversely, the topology recovered by the intron-set-based phylogenies was the fourth most common topology and occurred in 12% of the windows, while the exon-set-based MP-EST and ASTRAL topologies were the thirteenth and fourteenth most common topologies and appeared in only 2.5 and 2.1% of the windows, respectively (Fig. 4c). Altogether, the distribution of gene tree frequency in combination with short internal branches in the species tree is consistent with the expectation of the existence of an anomaly zone in Prunellidae.

### Effect of recombination rate variation on topology distribution

**Fig. 5:**
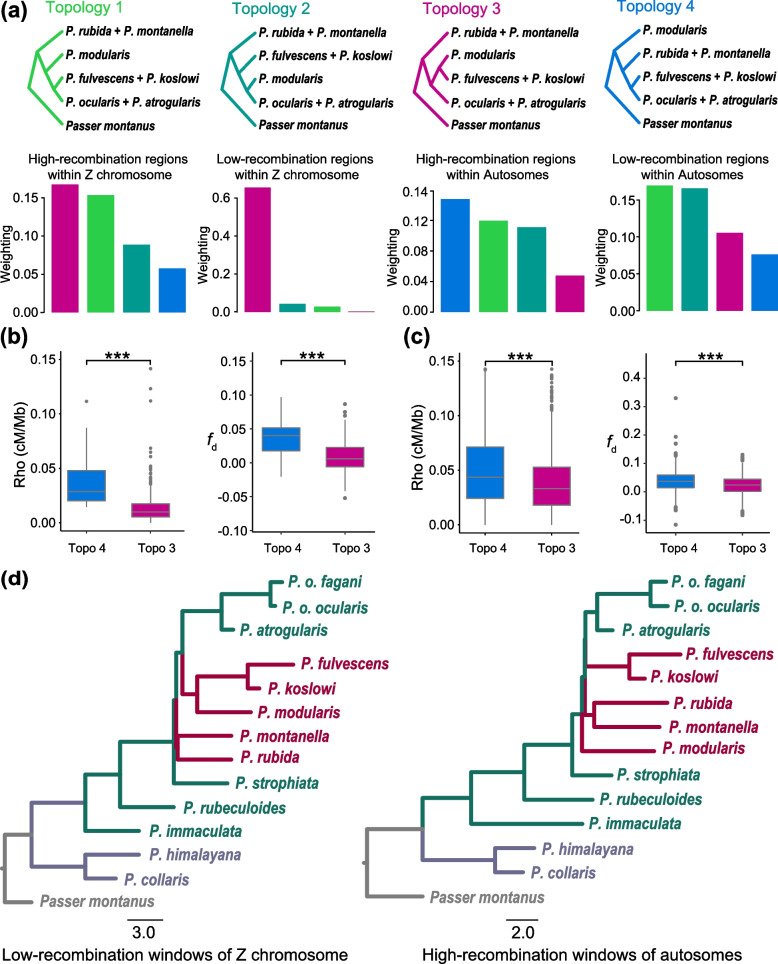
Tree topology changes with variation in recombination rate and introgression. **a** The frequency distribution of the four most common topologies in the high- and low-recombination regions of the autosomal and Z chromosomes, respectively. **b, c** Interplay between the topological distribution and recombination rate variation (left) as well as between the topological distribution and genetic introgression (right) in the Z chromosome (**b**) and autosomes (**c**). Topology 4 (blue), which is congruent with the phylogeny inferred from the intron-set, is enriched in the genomic regions with high-recombination rate and high level of gene flow, while the topology 3 (reddish) is more common in the genomic regions with low-recombination rates and less signature of gene flow. **d** ASTRAL species trees reconstructed for the low-recombination regions within the Z chromosome (left) and for the high-recombination regions within the autosomes (right), respectively. The two phylogenies differ in the position of *P. montanella*/*P. rubida*, *P. fulvescens*/*P. koslowi*, and *P. modularis* (indicated by reddish branches). The phylogeny of high recombination regions within autosomes is similar to those of intron-set

If the introgression is the predominant process generating topological discordance and anomaly zone, we would expect gene tree topology in the genomic regions with low recombination rate would be more resistant to introgression. We subsequently investigated tree topology and variation in introgression and recombination rates across the chromosomes for the species falling within the anomaly zone. We used population sequencing data from *P. modularis* (*n* = 9) to estimate recombination rates using ReLERNN [49] and PyRho v0.1.6 [50]. As the comparisons based on recombination rates estimated by ReLERNN and PyRho (see “Methods”) showed similar results, we present only the ReLERNN-based results in the main text; those based on PyRho are placed in the supplementary material (Additional file 1: Fig. S3). We averaged recombination rate (cM/Mb) in 50 kb non-overlapping windows and selected windows falling in the upper and lower 10% percentile of recombination rate and estimated topology distribution across these windows. We found that topology 4 ((*P. montanella*, *P. rubida*), ((*P. koslowi*, *P. fulvescens*), (*P. o. fagani*, *P. o. ocularis*, *P. atrogularis*))) was more frequent within the high-recombination regions of autosomes (Fig. 5a and Additional file 1: Fig. S3). This topology is congruent with phylogeny inferred from intron-set. In contrast, the low-recombination regions on the autosomes recovered topology 1 as having the highest frequencies. The analysis of the Z chromosome found topology 3 to be the dominant topology, especially in the low-recombination regions of that chromosome (Fig. 5a and Additional file 1: Fig. S3).

We then investigated the interplay between the topology distribution and variation in introgression and recombination rate. We specifically focused on gene flow between *P. modularis*, *P. ocularis*/*P. atrogularis*, *P. montanella*/*P. rubida*, and *P. fulvescens*/*P. koslowi* with *Passer montanus* as outgroup (see “Methods”). We found that the genomic regions supporting topology 4 have high rates of recombination and gene flow, while genomic regions supporting topology 3 have low rate of recombination rate and introgression (Wilcoxon statistic, *P* < 0.001, Fig. 5b and Fig. 5c, Additional file 1: Fig. S3 and Fig. S4). This pattern is more pronounced in the Z chromosome than in the autosomes.

We further reconstructed ASTRAL trees using 50-kb genomic windows with the upper and lower 10% percentile of recombination rate separately, and found that the topology from the genomic regions of the autosomes with the highest recombination rate was identical to the trees estimated from the intron-set-based phylogeny (Fig. 5d, Additional file 1: Fig. S4). However, the phylogenetic relationships reconstructed using the low-recombination regions in the Z chromosome placed *P. montanella* + *P. rubida* as a separate lineage, instead of clustering with *P. koslowi* + *P. fulvescens* as exhibiting by the phylogeny based on the high-recombination regions (Fig. 5d, Additional file 1: Fig. S4). Taken together, these results suggest that the low-recombination regions within the Z chromosome tend to contain few introgressed segments, likely representing the probable speciation-driven branching relationships for the accentors.

## Discussion

### Phylogenomic relationship of accentors

Lineages that have experienced a rapid radiation are prone to ILS and interspecific hybridization, a situation that poses a great challenge for phylogenetic reconstruction [6, 8]. The Prunellidae is a group of montane specialists that experienced a remarkably rapid cladogenesis during the Pliocene–Pleistocene [37, 38, 41]. By employing analyses of genome-wide intronic and exonic loci, we present the first whole genome phylogeny of this family. Similar to previous studies based on a small number of loci [37, 38], the phylogenomic analyses supported the division of the family into two major clades, *Laiscopus* and *Prunella*. These two groups of accentors show distinct ecological and morphological differences as *Laiscopus* consists of large alpine species and *Prunella* of small species associated with shrubby or forested habitats at generally lower elevations [51]. Due to these differences, the two groups have been proposed as distinct genera [37, 52]. Within the *Prunella* clade, *P. immaculata* and *P. rubeculoides* form two single-species lineages that are distantly related to the others. Their relationship relative to each other and to the remaining species of small accentors are also well supported in the topologies estimated using different analytical approaches and datasets.

In agreement with previous phylogenetic studies [37, 38], our phylogeny analyses found that the relationships among the remaining small *Prunella* accentors are poorly resolved. These small accentors were estimated to have diversified in a close succession at the Pliocene–Pleistocene boundary. This rapid radiation may have led to an occurrence of extensive ILS [41]. Indeed, the short internal branches within the *Prunella* clade observed in the coalescent and concatenated species trees are consistent with such the rapid radiation scenario and cause conflicting gene trees and uncertainty in phylogenetic reconstruction (e.g., [53–56]).

### Gene tree discordance and potential anomaly zone in accentor phylogeny

We observed that the species tree for Prunellidae had three successive short internal branches and that the most frequent gene tree topology detected in this region differed from that found in any estimate of the species trees. Taken together, these two observations suggest the presence of an anomaly zone in Prunellidae. This pattern is similar to situations where ILS alone produces an anomaly zone defined by two consecutive internal branches in an ancestor–descendant relationship in the species tree [57]. To date, only a few empirical examples of anomaly zones have been reported, and ILS has been regarded as the major reason for their occurrence (e.g., [7, 58, 59]). However, in the case of Prunellidae, coalescence simulations using the branch lengths observed in the MP-EST and ASTRAL species trees suggest that at most 44–75% of the gene tree discordance is likely attributable to coalescent variation arising from ILS and gene tree estimation errors (i.e., under high ILS and gene tree estimation errors). These results therefore suggest that ILS and gene tree estimation errors cannot explain the observation of the three successive short internal branches.

Our results also detect extensive genetic introgression among the four lineages stemming from the central part of the tree, which exacerbates and complicates the interpretation of the anomaly zone. The combination of ILS and gene flow maximizes gene tree variation and distortion of gene tree distributions, and the effects of one process on gene tree landscapes can mimic the effects of the other [6]. Previous simulation studies show that gene flow alone can create anomalous gene trees and produce an anomaly zone (e.g., [14, 15]). However, the interaction of gene flow, effective population size (i.e., the coalescence rate) in the recipient species and the lengths of speciation interval (i.e., coalescent time) are not simple and can interact in complicated ways to affect the species tree [15]. As such, our study suggests a new empirical challenge to the study of the anomaly zone: how can we identify the source of anomaly zones in the presence of gene flow? Thus, we only cautiously extend our inference of an anomaly zone to the case of the Prunellidae, because the signatures of an anomaly zone depend on the interaction between the migration rate, the population sizes, and the lengths of the speciation intervals [15]. Future work will be needed for find ways to disentangle the effects of gene flow on gene tree topologies and the anomaly zone.

### Effects of variation in genetic introgression and recombination rate on topology distribution

For lineages with an extensive history of hybridization, the prevailing phylogenetic signal within the autosomes may not always be representative of the most probable speciation history [10, 11, 31]. Indeed, our findings indicate that the phylogenetic signal is not randomly distributed across the chromosomes but is strongly structured by variation in recombination rate and retention of introgressed segments. For example, a tree that is characterized by genetic introgression (i.e., great *f*_d_ values) is enriched in windows with high-recombination rates, while a tree with few introgression segments (i.e., low *f*_d_ values) is more commonly found in the low-recombination regions within the Z chromosome. Nature selection affects recombination rate that further influence how genealogical histories are distributed across chromosomes, as introgressed alleles are more likely to persist within high-recombination regions than within low-recombination regions, because neutral or positively selected variants may be effectively unlinked from deleterious alleles in high-recombination regions, and hence less likely to be removed as a result of background selection [34, 35]. Our study corroborates previous studies suggesting that low-recombination regions are more likely to reflect the original speciation events in the presence of gene flow than high-recombination regions [11, 60, 61].

Our study also corroborates earlier suggestions that, in birds, the Z chromosome may represent the species tree better than autosomes (e.g., [62]). This is consistent with the “large X/Z-effect” identified in mosquitoes [61], cats [11], and birds [63–65]. For example, it was found that Z-linked single-nucleotide polymorphism (SNP) markers showed little evidence of introgression in hybridizing Old World *Ficedula* flycatchers, whereas substantial introgression was documented for autosomal SNPs [63]. This pattern stems in part from sex chromosomes tending to be enriched for genetic elements with large effect on reducing hybrid reproductive fitness and is thus more likely to track ancient speciation events [10, 35, 60–62]. Taken together, our results demonstrate that diversification in the presence of gene flow can create a phylogenomic architecture where the most accurate depiction of the phylogenetic tree persists only within the small fraction of the genome that possesses historically reduced rates of recombination.

### Potential limitations of this study

One limitation of the current work is how we can confidently detect an empirical anomaly zone that may stem from ILS and gene flow. In our study, we used the equation in Degnan and Rosenberg [12] to define the anomaly zone. This approach assumes non-existence of gene flow between lineages after speciation. In fact, gene flow between distantly related lineages can produce high levels of homoplasy. This will increase the coalescent time of these lineages and decrease internal branch lengths [11]. This process may have an effect of creating successive, short internal branches similar to those observed in an anomaly zone produced by ILS, especially when multiple episodes of hybridizations occur during radiations. Here we also estimated gene flow anomaly zone using quartet concordance factors as described in Solís-Lemus et al. [14] and Long & Kubatko [15], as well as estimated topology frequency distribution across the chromosomes. Still, we do not know how ILS and gene flow influence this anomaly zone. Investigations of the empirical anomaly zone should benefit greatly from future theoretical work that clarifies the relative influences of ILS and gene flow.

## Conclusions

Evaluation of the processes generating observed patterns of gene tree discordance is still in its infancy. In this study, by using genome-wide sequencing, we created rich and dense datasets to tease apart the alternative hypotheses about the sources of topological conflicts in the phylogeny of Prunellidae. We show that the observed topological incongruences mainly stem from three successive internal nodes falling into an anomaly zone, where incomplete lineage sorting and genetic introgression mislead gene tree topologies. This anomaly zone carries clear implications for the phylogenetic inference as the most commonly observed gene tree topology may differ from that of the true species tree. The multispecies coalescent methods model gene trees as conditionally independent variables and can thus accurately infer the species tree under high levels of ILS, and in extreme case even in the presence of an anomaly zone [66, 67]. However, if genetic introgression has been the major force to produce such an anomaly zone, applying standard phylogenomic approaches may infer ancestral speciation events incorrectly [11, 68]. Our study of the Prunellidae, together with those of other species (i.e., cats [11]; butterflies [10]; mosquitoes [61]), have demonstrated how genetic introgression has influenced chromosome-wide patterns of phylogenetic variation. Given this, prior knowledge about genome architecture, specifically introgression and recombination rate variation, should be considered in future phylogenomic analyses [69].

## Methods

### De novo genome assembly of Prunella strophiata

A male of *Prunella strophiata* (Voucher ID XZ15142) collected from Linzhi, Tibet (Lat. 29.39, Lon. 94.42, Elev. 3,880 m), was used for de novo genome sequencing. Genomic DNA from muscle was extracted using the standard phenol/chloroform extraction method. We used the PacBio SEQUEL II platform and the Illumina NovaSeq platform for genomic sequencing. A PacBio library was constructed using the Pacific Biosciences SMRTbell Template Prep Kit and sequenced on a Pacific Biosciences Sequel II platform in Annoroad Gene Technology (Beijing). Data for short reads (150-bp paired-end) were generated using an Illumina NovaSeq platform.

The PacBio long reads were initially assembled with Canu package v1.8 [70] and Wtdbg (https://github.com/ruanjue/wtdbg). The obtained draft assembly was then refined using long reads with Arrow [71] and short reads with Pilon [72]. We used the Redundans [73] to remove redundant scaffolds. The completeness and accuracy of the genome assembly were assessed via short-read mapping and Benchmarking Universal Single-Copy Orthologs (BUSCO) analysis. We further assembled the contigs into chromosome-level scaffolds using Hi-C technology on the same individual. The Hi-C library was prepared and sequenced using the Illumina HiSeq platform with 2 × 150-bp reads at BGI-Shenzhen. Hi-C reads were then mapped to the contigs using JUICER v1.6.2 [74] and 3D-DNA v190716 [75] was used to anchor contigs to scaffolds. Possible assembly errors such as misjoins, translocations, and inversions were manually examined and corrected using the Assembly Tools module within JUICEBOX v1.11.08 [74] (Additional file 1: Fig. S5). We aligned *P. strophiata* genome with the Zebra finch (*Taeniopygia guttata*) genome using MUMmer v3.23 [76] and checked the collinearity of the two genomes.

### Taxon sampling

We included all currently recognized species of Prunellidae (Supplementary Table 1), which consists of a single genus (*Prunella*) with twelve species [40, 42]. *Prunella ocularis fagani* was previously treated as a distinct species [77] but is now treated as a subspecies of *P. ocularis* [40, 42]. As *P. o. fagani* is geographically widely separated from *P. o. ocularis*, we herein treat *P. o. fagani* and *P. o. ocularis* as two taxonomic units. We included two to nine individuals for each species except for *P. koslowi* and *P. atrogularis*, for which only a single individual was available for each species. We used cryo-frozen or 96% ethanol-preserved tissue for all taxa except for *P. o. fagani* for which DNA was extracted from the toepad of a museum study skin.

### DNA extraction, library preparation, and resequencing

The DNA was extracted from the tissue and museum toepad samples of 34 accentors and two Tree Sparrow *Passer montanus* using the Qiagen QIAamp DNA Mini Kit according to the manufacturer’s protocol. Sequencing libraries for fresh tissues were prepared using the Illumina TruSeq PCA-free (190/350 bp) kit and were sequenced on an Illumina Novaseq platform in Annoroad Gene Technology and Berry Genomic Institute. The library from museum specimen was prepared using the protocol published by Irestedt et al. [78] and sequenced by SciLifeLab (Stockholm). The samples were sequenced to a mean coverage of 21 × (Supplementary Table S2).

### Filtering raw reads and reference mapping

Raw sequenced data were cleaned using the fastx toolkit (http://hannonlab.cshl.edu/fastx_toolkit/) with the following steps: (1) removal of adapters, (2) removal low-quality reads; reads with the proportion of “N” > 3% or reads with > 50% low-quality bases (< 3). Raw sequencing data from the museum specimen were cleaned by the same procedure except deleting 5 bp from both ends to avoid wrong sequences of the degraded DNA. We mapped clean reads of 34 accentors, and two tree sparrow*s* and one Red-banded flowerpecker ( *Dicaeum eximium*, GCA013396995) against the de novo genome of *P. strophiata* using BWA mem v0.7.12 [79], and then sorted and removed duplicates using Picard (http://broadinstitute.github.is/picard/). We called variants using bcftools mpileup v1.9 [80]. We removed indels and filtered variant call format (VCF) using criteria: (1) minQ > 30, (2) min-DP > 10 and max-DP < 2500, (3) max-missing rate ≤ 0.1, (4) SNPs at least 5 bp away from indels. The VCF after filtering was used for downstream analysis.

### Extracting and aligning homologous exonic and intronic loci

To investigate the potential influence of different genetic markers on phylogenetic inference, we assembled intronic and exonic datasets. We carried out these steps using a custom designed BirdScanner pipeline [81] (github.com/Naturhistoriska/birdscanner). Specifically, we performed searches using profile hidden Markov models (HMM) [82] to obtain a large number of sequence homologs of nuclear exonic and intronic loci across the whole genome. Profile HMMs use information from variation in multiple sequence alignments to seek similarities in databases, or as here, genome assemblies [83]. The HMM profiles were based on the alignments of exonic and intronic loci generated by Jarvis et al. [1] for four passerine species, *Acanthisitta chloris*, *Corvus brachyrhynchos*, *Geospiza fortis*, and *Manacus vitellinus*. For each HMM query and taxon, the location in the genome for the highest hit was identified, and the sequence parsed out using the genomic coordinates. The parsed-out gene sequences were then aligned gene by gene using MAFFT v7.310 [84] and poorly aligned sequences were identified, based on a calculated distance matrix using OD-Seq (github.com/PeterJehl/OD-Seq) and excluded from further analyses. We also checked the alignments manually and removed those that included non-homologous sequences for some taxa (indicated by an extreme proportion of variable positions in the alignment) and those that contained no phylogenetic information (no parsimony-informative sites). We also filtered the alignments to only include those that contained all samples. A total of 2373 exonic and 6879 intronic loci were kept for the subsequent analyses. All separate alignments were combined to a single concatenated alignment for the concatenation analyses, or kept separate for coalescent analyses based on gene trees.

### Phylogenomic analyses

We used both concatenated and coalescent approaches to estimate phylogenomic relationships of the accentors for the intron-set and exon-set, respectively. For the concatenated approach, trees were constructed for the exon-set and intron-set separately using IQ-TREE [43] and applying “–m TEST” option to find the best substitution model for each alignment. We inferred the maximum-likelihood trees from the two concatenated datasets with 1000 ultrafast bootstraps to obtain branch supports as implemented in the IQ-TREE software [85].

For the coalescent analyses, we first used IQ-TREE to estimate the best maximum-likelihood tree for each intronic or exonic dataset. Statistical confidence of each gene tree was assessed by performing 100 bootstrap replicates using the best substitution model for each alignment. We used ASTRAL-III v5.6.3 [44, 45] to construct coalescent trees from the best maximum-likelihood gene trees estimated for the exon-set and intron-set separately. We also ran MP-EST coalescent analyses (MP-EST v2.1) [46] with 100 runs beginning with different random seed numbers and ten independent tree searches within each run. The MP-EST species tree topology was inferred using the best maximum-likelihood gene trees as input. Confidence of each node was evaluated by performing the same species tree inference analysis on 100 maximum-likelihood bootstrap gene trees. The resulting 100 species trees estimated from bootstrapped samples were summarized onto the ASTRAL and MP-EST species trees using the option “-f b” in RAxML.

### Test topological difference between estimated gene trees and species trees

We next considered whether topological differences between estimated gene trees and the species trees are well supported. For each locus, we tested the estimated gene tree topology against each of the four candidate species trees that were inferred for the intron-set and exon-set, respectively (see “Results”). We used approximately unbiased (AU) tests in IQ-TREE to test whether individual gene trees fit each of the four candidate species trees. For each gene tree, a Bonferroni-corrected *P* value of 0.05 adjusted for multiple comparisons was considered to reject species tree topology.

### Coalescent simulations

To investigate how much gene tree heterogeneity can be explained by ILS and gene tree estimation error, we carried out coalescent simulations as described in Cai et al. [6]. For the intron-set and exon-set, we estimated the ultrametric species tree branch lengths in mutational units ($\upmu T$, where $\upmu$ is the mutation rate per generation and *T* is the number of generations) by constraining the concatenated alignments of all loci to each of four species trees with a GTR + GAMMA substitution model and strict molecular clock in PAUP. These mutational branch lengths from the constrained tree and branch lengths in coalescent units ($\uptau ={\rm T}/4N\mathcal{e}$) from MP-EST and ASTRAL species trees were used to estimate the population size parameter theta ($\Theta$) for each internal branch following Degnan and Rosenberg [12]. To simulate conditions of high and low levels of ILS, we modified theta values when generating gene trees under the coalescent model using the function “sim.coaltree.sp” in Phybase v1.5 [86]. Theta value is positively correlated with gene tree discordance. Here we set theta value of 0.001 and 0.1 (corresponding to the span between the minimum and maximum theta values observed in the empirical datasets) to reflect low and high ILS. We simulated 1500 gene trees from each of the species trees for intron-set and exon-set, respectively.

From each of the simulated gene trees, we used AliSim [87] in IQ-TREE to simulate alignments to reflect gene tree estimation error stemming from intron-set and exon-set, respectively. We generated sequence alignments of 500 bp in length for the exons and 1000 bp for the introns (these lengths correspond to the mean lengths of the respective set of empirical gene alignments). We applied the GTR + I + G model for nucleotide substitutions with the parameter values observed when optimizing the concatenated exon and intron datasets to a constrained tree topology (i.e., the respective MP-EST species tree) in RAxML. Gene trees were then estimated using IQ-TREE as described above. The simulated gene trees, empirical gene trees, and species trees have same number of taxa, i.e., 37 taxa.

We calculated pairwise RF distances and matching cluster distances of each simulated gene tree to the species tree topology using TreeCmp [88]. The ratio of mean gene tree to species tree distance for the simulated gene trees relative to mean distances for the empirically estimated gene trees was calculated as a measure of the amount of observed gene tree discordance that can be accounted for by ILS and gene tree estimation errors.

### Analysis of introgression

We used three methods to identify introgression among the seven species that showed the greatest extent of topological disparity. Here we treated *P. o. ocularis* and *P. o. fagani* individually as these taxa are widely separated geographically. First, we calculated Patterson’s *D*-statistics to assess introgression using the ABBA-BABA test as implemented in the program Dsuite [89]. This test is applied to biallelic SNPs across four taxa and assumes a tree topology typically given as (((P1, P2), P3), O). The outgroup (O) helps to polarize the ancestral allele (A) from the derived allele (B) and site patterns (BBAA, ABBA, and BABA, respectively) are counted. Under the assumption of absence of deviation from a strict bifurcating topology and an equal mutation rate, we expect to observe roughly equal proportions of ABBA and BABA patterns in the genome. A significant deviation from this suggests presence of introgression between P3 and either P1 or P2 [89]. We assessed all possible trios of the eight taxa using the *Passer montanus* as outgroup, in order to explore the potential gene flow between any species pairs, without setting any prior topology. We used standard block-jackknife procedure to estimate an overall combined *P* values for *D* statistics across all genomic regions. Specifically, block-jackknife estimates the standard deviation for so-called “pseudo-values” of the mean genome-wide *D*, where each pseudo-value is computed by excluding a defined block of the genome, taking the difference between the mean genome-wide *D* and *D* computed where the block is omitted. We used a block size of 1 Mb as recommendation in [89]. To account for multiple tests, *P* values were corrected by the false discovery rate (FDR) [90].

Second, we estimated the *f*-branch metric (fb) [91] using Dsuite. The *f*_b_ statistic assigns gene flow to specific, possible internal branches on a phylogeny as described in Martin et al. [92]. By using the topology inferred from the low-recombination regions of the Z chromosome as the likely species tree (see “Results”), we estimated the *f*_b_ (P3) statistic as median_*A*_ [min_*B*_[f4ratio (*A*,*B*;*P3*,*O*)]], where *B* refers to the populations or taxa that are descendants of *b*, and *A* refers to descendants of *b*’s sister branch α. The *f*_4_ ratio (*A*,*B*;*P3*,*O*) reflects the relative excess sharing of alleles between the descendants of branch *b* (*B*) and species *P3*, compared with allele sharing of the descendants of the sister branch α (*A*) and *P3*, under the assumption of equal mutation rate. The min_*B*_ takes the minimum from *B*, the species descending from branch *b*, and the median_*A*_ takes the median across descendants of *b*’s sister branch α [91]. We used Dtrios to estimate *f*_4_ ratio statistics for all trios and used Fbranch to calculate Dtrios results and *f*-branch statistic. The calculations were carried out with all positive *f*_4_ ratio results that had A in the P1 and B in the P2 positions. Each *f*_b_(P3) score was assigned an associated Z-score to assess statistical significance. In both analyses, we used *Passer montanus* as the outgroup to avoid any confounding influence that may stem from potential gene flow between any two accentors.

Third, we used five-taxon comparisons performed with *D*_FOIL_ [93] to quantify the ancient ingrogression as indicated by the unique signatures of interlineage introgression that are consistently retained in each descendant species. We focused on species combinations that could test episodes of ancestral introgression, i.e., interlineage introgression. We used chi-square goodness-of-fit test and a threshold of *P* < 0.001 to identify significant ancestral introgression events [9, 93].

### Test of the anomaly zone

Without gene flow, the anomaly zone occurs where a set of short internal branches in the species tree produce gene tree that differs from the species tree more frequently than the tree that is concordant, described as *a*(*x*), as defined in Eq. 4 of Degnan and Rosenberg [12]. To explore if gene tree discordance observed in Prunellidae is in principle a product of the anomaly zone, we calculated the value *a*(*x*) for each internal branch *x* that measures in coalescent unit for MP-EST and ASTRAL species trees. The calculated *a*(*x*) value was compared to the coalescent length for each descendent internal branch *y*, where *y* < *a*(*x*) provides evidence that this region of the species tree falls within the anomaly zone where the anomalous gene tree topology is most numerous among the gene trees. We also estimated the concordance and discordance factors (proportions of gene trees that are concordant or discordant with the species tree) to estimate anomaly zone in presence of gene flow as described in Solis-Lemus et al. [14] and Long and Kubatko [15]. We calculated gene concordance factor (gCF) and discordance factor (gDF) implemented in IQ-TREE to measure the frequency of gene trees that are concordant or discordant with putative species trees, respectively [94]. The gCF is the proportion of input gene trees decisive for a particular branch from a species tree and gDF measures the proportion of gene trees that result in alternative topologies [94]. Using the MP-EST and ASTRAL species trees as reference, gCF and gDF were computed across all nodes of the species tree and gene trees.

### Polytomy test

Huang and Knowles [47] pointed out that the gene tree discordance produced from the anomaly zone can also be generated by uninformative gene trees and that for species tree with short branches the most probable gene tree topology is a polytomy rather than an anomaly zone. We therefore performed a polytomy test in ASTRAL as described in Sayyari and Mirarab [95] to evaluate whether the short successive internal branches in the *Prunella* clade could have been generated by polytomy. We used the MP-EST and ASTRAL species trees as the guide trees and analyzed the gene trees generated from the intron-set and exon-set separately.

### Topology distribution across the chromosomes

We used TWISST [48] to examine topology distribution across the chromosomes. We specifically investigated the relationships among the four lineages that were connected by three successive internal branches (i.e., *P. modularis*, *P. rubida*/*P. montanella*, *P. fulvescens*/*P. koslowi*, and *P. ocularis*/*P. atrogularis*; see “Results”) with *Passer montanus* as an outgroup. We followed the pipeline of Martin and van Belleghem [48] (available at: https://github.com/simonhmartin/genomics_general/) to investigate the distribution of topologies across the chromosomes using a 50-kb sliding window. The proportions of windows supporting the different topologies were calculated as topology weight and the results were visualized using scripts provided on the TWISST github page.

### Estimating recombination rates

We used population sequencing data from *P. modularis* (*n* = 9) to estimate recombination rates using ReLERNN [49] and PyRho v0.1.6 [50]. ReLERNN leverages recurrent neural network by using the raw genotype matrix as a feature vector. This method avoids converting the data into summary statistics and is thus robust to small numbers of sequenced genomes. We first estimated the population demography using SMC +  + [96] and set the estimated demographic parameters to –demographicHistory to account for the change of population history [96]. ReLERNN calculated *r* (recombination rate per base pair per generation) by simulating data matching the theta value of the observed DNA sequences (“ReLERNN_SIMULATE” function). Simulations were then used to train and test a recurrent neural network model designed to predict the per-base recombination rate (“ReLERNN_TRAIN” function). We subsequently predicted per-base recombination rates across chromosomes (“ReLERNN_PREDICT” function).

For PyRho, we followed practices as recommended (https://github.com/popgenmethods/pyrho). This program uses a composite-likelihood approach to infer recombination rate from individual polymorphism data. The genotype data are used to compute a lookup table of two-locus likelihood of linkage disequilibrium, which is used to set up the hyperparameters of the model. An innovative feature of PyRho is that the lookup table accounts for demographic change by using independent estimates of effective population size fluctuation over time when computing the two-locus likelihood. We estimated population demographic parameters using SMC +  + and provided the output using the –smcpp-file option. We estimated recombination rate across the chromosomes using the parameters determined from the pyrho_hyperparam function, while other parameters were left with default settings.

To assess how support for a specific topology (inferred from TWISST analyses) varies with recombination rate, we transformed the outputs from ReLERNN and PyRho to average recombination rate (cM/Mb) in 50-kb non-overlapping windows. We selected windows falling in the upper and lower 10% percentile of recombination rate and estimated topology distribution across these windows.

### Integrating signals of topology distribution and variation in introgression and recombination rate

We estimated chromosome-wide introgression using *f*_d_ statistic in 50-kb non-overlapping sliding windows using ABBABABAwindows.py [97]. We specifically focused on comparisons between *P. montanella*/*P. rubida* and *P. fulvescens*/*P. koslowi* as these two lineages constitute the major topological conflicts observed (see “Results”). We estimated *f*_d_ values for each of the four trios using *P. modularis* as outgroup, and then calculated their average *f*_d_ values for subsequent comparisons. To assess how topology frequency changes with variation in introgression and recombination rate, we compared average *f*_d_ and *r* values between the windows supporting the different topologies for the autosomal and Z chromosomes, respectively. We used Wilcoxon statistic to test for statistical significance.

### Supplementary Information

**Additional file 1: Fig. S1.** Synteny of aligned *P. strophiata* genome with zebra finch genome and these two genomes showed high collinearity. **Fig. S2.** Polytomy test for the MP-EST and ASTRAL species trees as the guide trees. **Fig. S3.** Tree topology weights vary with recombination rate (estimated from PyRho). **Fig. S4.** Interplay between topology and variation in introgression rate. **Fig. S5.** Hi-C heatmap reconstructed for *Prunella strophiata* genome. **Table S1.** Statistics of the assembly of *Prunella strophiata *genome. **Table S2.** Completeness of the genome assembly of *Prunella strophiata *evaluated by BUSCO. **Table S3.** Chromosome synteny of aligned Red-breasted accentor genome with zebra finch genome. **Table S4.** List of the species were used for phylogenetic analyses. **Table S5.** Resequencing information and genome wide coverage of 36 individuals used in this study. **Table S6.** Gene concordance factor (gCF) for the nodes (1–7, Fig. 4a-b) that support the species tree (gCF), the two most common alternative topologies (gDF1 and gDF2), and the relative frequency of all other topologies (gDFp).

## Data Availability

Variant call data, alignments, and trees of intron and exon, and codes for this study are available in the figshare repository (https://doi.org/10.6084/m9.figshare.25202057.v3) [98]. Resequencing data of 34 accentors are available in the NCBI database under accession number PRJNA960939 (https://www.ncbi.nlm.nih.gov/bioproject/PRJNA960939) [99]. Genome assembly of *Prunella strophiata* is available in GenBank (https://www.ncbi.nlm.nih.gov/nuccore/JAZBQD000000000) [100].

## Abbreviations

ISL: Incomplete lineage sorting
TWISST: Topology weight analysis
gCF: Gene concordance factor
gDF: Gene discordance factor
RF: Robinson-Foulds
HMM: Hidden Markov models
BS: Bootstrap support
