# Supplementary material for: Gene flow and an anomaly zone complicate phylogenomic inference in a rapidly radiated avian family (Prunellidae)
Source: BMC Biol. 2024 Feb 27;22:49. doi: 10.1186/s12915-024-01848-7 (PMC10900574; doi:10.1186/s12915-024-01848-7)
Supplement: Supplementary file 1 — Additional file 1: Fig. S1. Synteny of aligned P. strophiata genome with zebra finch genome and these two genomes showed high collinearity. Fig. S2. Polytomy test for the MP-EST and ASTRAL species trees as the guide trees. Fig. S3. Tree topology weights vary with recombination rate (estimated from PyRho). Fig. S4. Interplay between topology and variation in introgression rate. Fig. S5. Hi-C heatmap reconstructed for Prunella strophiata genome. Table S1. Statistics of the assembly of Prunella strophiata genome. Table S2. Completeness of the genome assembly of Prunella strophiata evaluated by BUSCO. Table S3. Chromosome synteny of aligned Red-breasted accentor genome with zebra finch genome. Table S4. List of the species were used for phylogenetic analyses. Table S5. Resequencing information and genome wide coverage of 36 individuals used in this study. Table S6. Gene concordance factor (gCF) for the nodes (1–7, Fig. 4a-b) that support the species tree (gCF), the two most common alternative topologies (gDF1 and gDF2), and the relative frequency of all other topologies (gDFp). [file 12915_2024_1848_MOESM1_ESM.docx]

Supplementary Information

Additional File 1

Fig. S1. Synteny of aligned *P. strophiata* genome with zebra finch genome and these two genomes showed high collinearity.


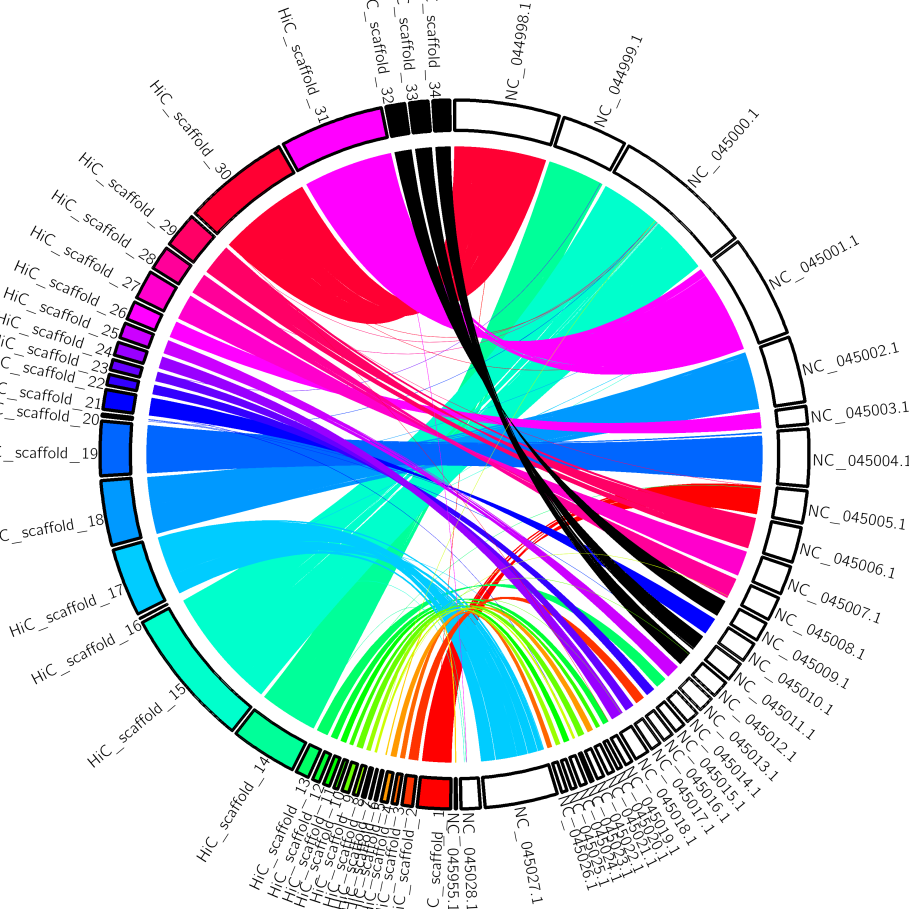


Fig. S2. Polytomy test for the MP-EST and ASTRAL species trees as the guide trees. Left two gene trees generated from intronic loci, and right two gene trees generated from exonic loci. The orange squares in the two MP-EST and ASTRAL species trees based on exon-set show the three polytomies but not in those based on intron-set.


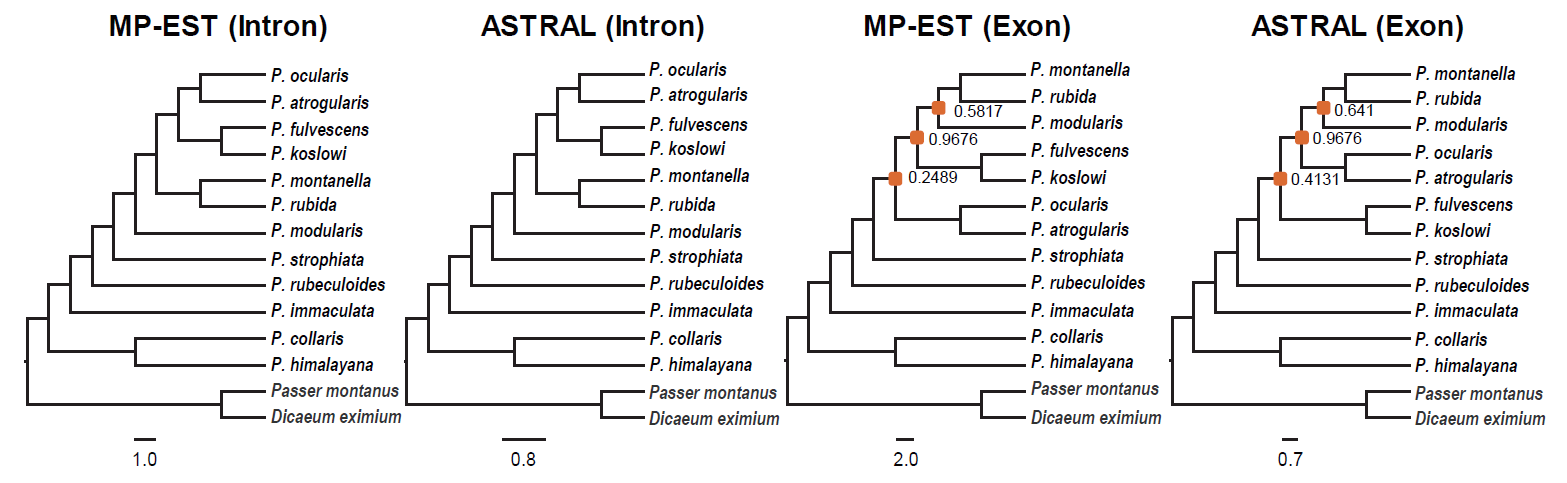


Fig. S3. Tree topology weights vary with recombination rate (estimated from PyRho). (a) The frequency distribution of the four most common topologies in the high- and low-recombination regions of the autosomal and Z chromosomes, respectively. (b) and (c) Interplay between topology distribution and recombination rate variation (left) as well as between topology distribution and genetic introgression (right) in the Z chromosome (b) and autosomes (c). Topology 4 (blue), which is congruent with the phylogeny inferred from the intron-set, is enriched in the genomic regions with high-recombination rate and high level of gene flow, while the topology 3 (ruby) is more common in the genomic regions with low-recombination rates and less signature of gene flow. (d) ASTRAL species trees are reconstructed for the low-recombination regions within the Z chromosome (left) and for the high-recombination regions within the autosomes (right). The two phylogenies differ in the position of *P. montanella*/*P. rubida*, *P. fulvescens*/*P. koslowi* and *P. modularis* (indicated by red branches). The phylogeny of high-recombination regions within autosomes is similar to those of intron-set by clustering *P. montanella*/*P. rubida* and *P. koslowi*/*P. fulvescens* together.


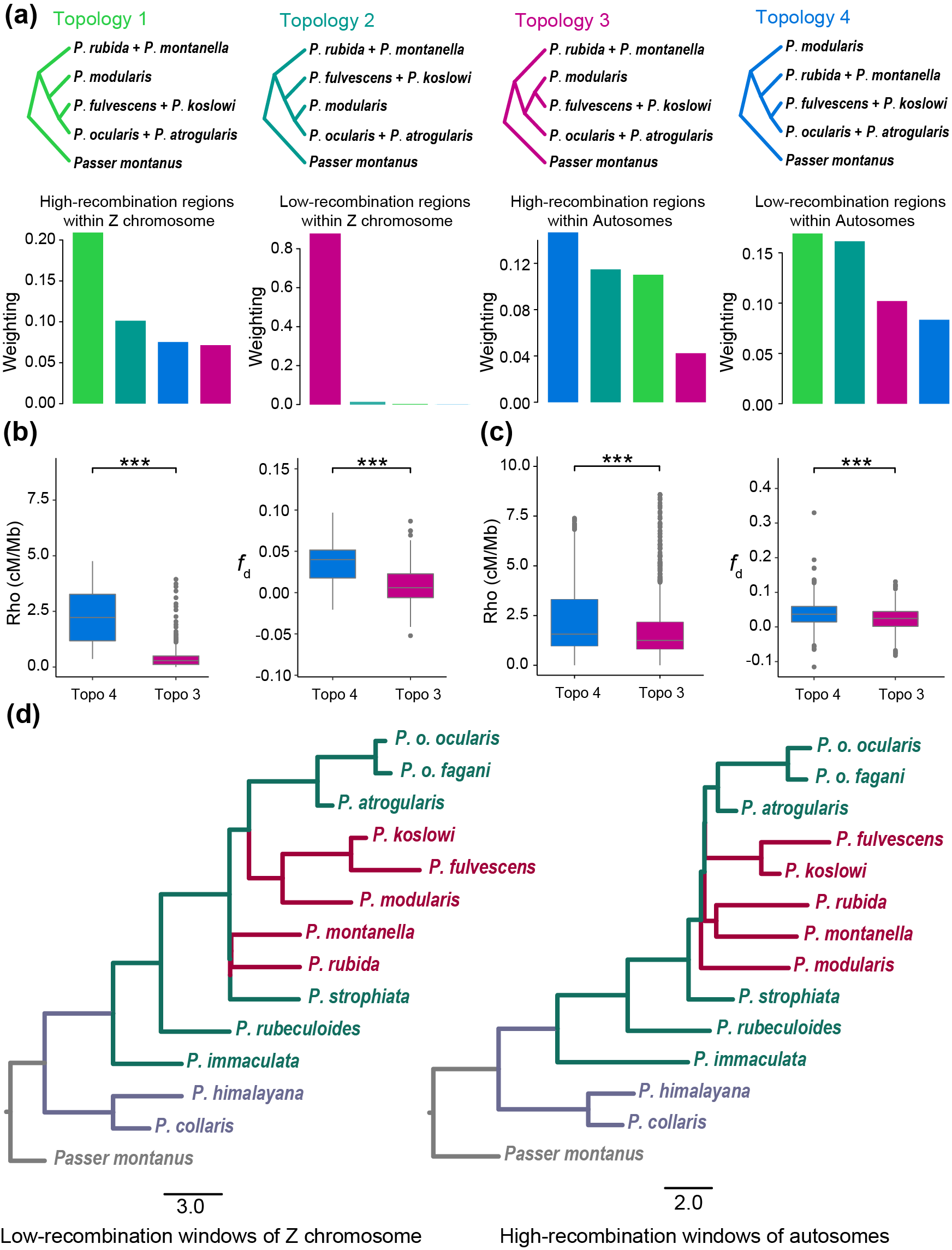


Fig. S4. Interplay between topology and variation in introgression rate. The two topologies (topology 4 and 3) of the node P-3 and signatures of introgression (*f*_d_ values estimated in 50-kb sliding window) from each of trois of *P. montanella*, *P. rubida*, *P. fulvescens* and *P.* *koslowi*. Topology 4 is more commonly found in the genomic regions with high levels of gene flow than the topology 3.


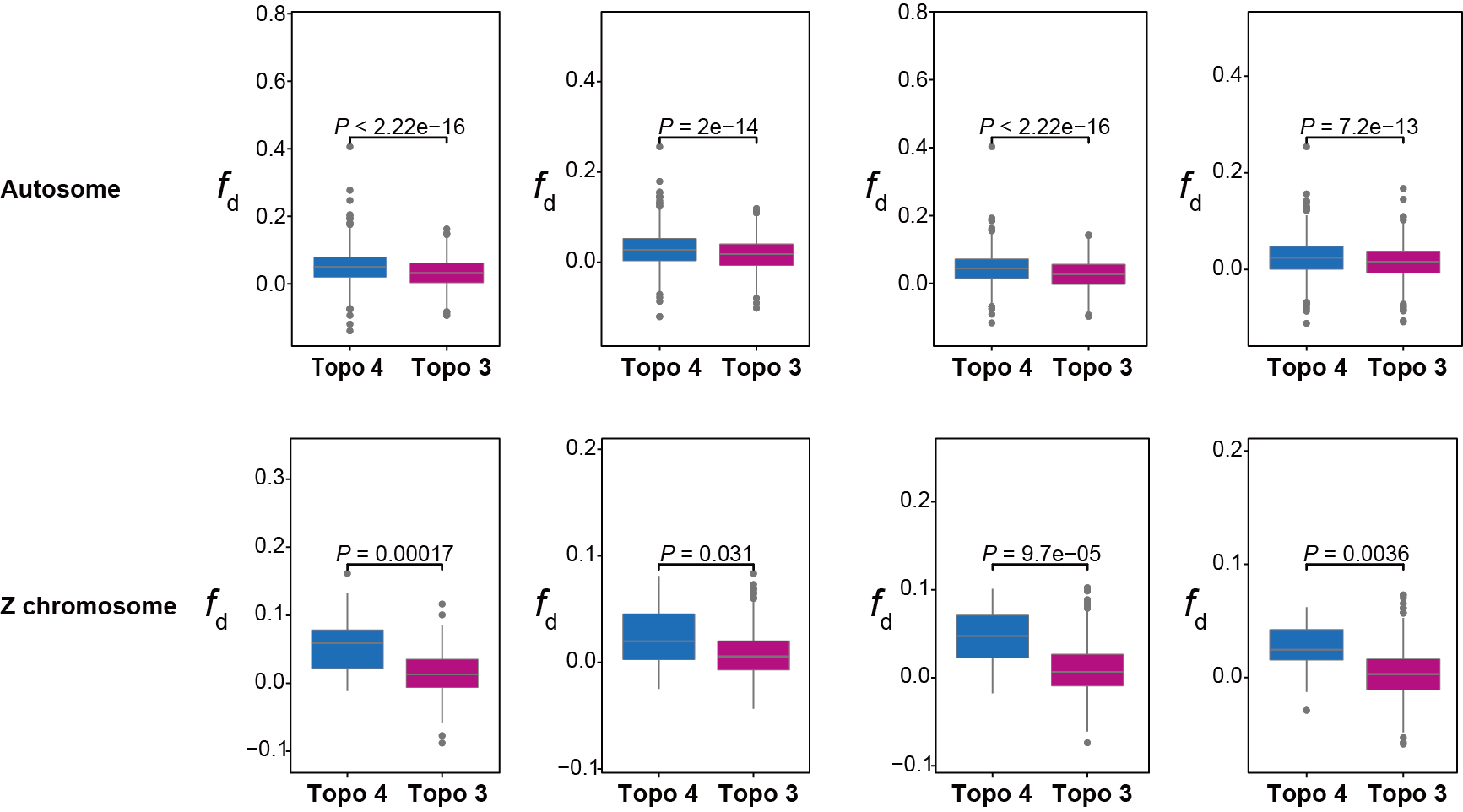


Fig. S5. Hi-C heatmap reconstructed for *Prunella strophiata* genome.

Table S1. Statistics of the assembly of *Prunella strophiata* genome.

| Stat Type | Contig length (bp) | Contig Number |
| --- | --- | --- |
| Total | 1055147219 | 2,530 |
| Max length | 52537528 | - |
| Number>=2kb | - | 2,528 |
| N50 | 9,754,116 | 27 |
| N60 | 5,900,415 | 41 |
| N70 | 3,555,124 | 64 |
| N80 | 1,129,363 | 117 |
| N90 | 232,380 | 332 |

Table S2. Completeness of the genome assembly of *Prunella strophiata* evaluated by BUSCO.

| I-terms | Number | Percent (%) |
| --- | --- | --- |
| Complete BUSCOs (C) | 4,401 | 89.6% |
| Complete and single-copy BUSCOs (S) | 4,333 | 88.2% |
| Complete and duplicated BUSCOs (D) | 68 | 1.4% |
| Fragmented BUSCOs (F) | 299 | 6.1% |
| Missing BUSCOs (M) | 215 | 4.3% |
| Total BUSCO groups searched | 4,915 | 4.3% |

Table S3. Chromosome synteny of aligned Red-breasted accentor (*Prunella strophiata*) genome with zebra finch (Taeniopygia guttata) genome.

| Chromosomes of *P. strohiata* | Chromosomes of Zebra finch | Chromosomes |
| --- | --- | --- |
| HiC_scaffold_1 | NC_045005.1 | Autosome |
| HiC_scaffold_2 | NC_045016.1 | Autosome |
| HiC_scaffold_3 | NC_045026.1 | Autosome |
| HiC_scaffold_4 | NC_045021.1 | Autosome |
| HiC_scaffold_5 | NC_045955.1 | Autosome |
| HiC_scaffold_6 | NW_022611545.1 | Autosome |
| HiC_scaffold_7 | NC_045023.1 | Autosome |
| HiC_scaffold_8 | NC_045025.1 | Autosome |
| HiC_scaffold_9 | NC_045022.1 | Autosome |
| HiC_scaffold_10 | NC_045020.1 | Autosome |
| HiC_scaffold_11 | NC_045024.1 | Autosome |
| HiC_scaffold_12 | NC_045019.1 | Autosome |
| HiC_scaffold_13 | NC_045014.1 | Autosome |
| HiC_scaffold_14 | NC_044999.1 | Autosome |
| HiC_scaffold_15 | NC_045000.1 | Autosome |
| HiC_scaffold_17 | NC_045027.1 | Z chromosome |
| HiC_scaffold_18 | NC_045002.1 | Autosome |
| HiC_scaffold_19 | NC_045004.1 | Autosome |
| HiC_scaffold_20 | NC_045012.1 | Autosome |
| HiC_scaffold_21 | NC_045010.1 | Autosome |
| HiC_scaffold_22 | NC_045015.1 | Autosome |
| HiC_scaffold_23 | NC_045017.1 | Autosome |
| HiC_scaffold_24 | NC_045018.1 | Autosome |
| HiC_scaffold_25 | NC_045013.1 | Autosome |
| HiC_scaffold_26 | NC_045003.1 | Autosome |
| HiC_scaffold_27 | NC_045007.1 | Autosome |
| HiC_scaffold_28 | NC_045008.1 | Autosome |
| HiC_scaffold_29 | NC_045006.1 | Autosome |
| HiC_scaffold_30 | NC_044998.1 | Autosome |
| HiC_scaffold_31 | NC_045001.1 | Autosome |
| HiC_scaffold_32 | NC_045009.1 | Autosome |
| HiC_scaffold_33 | NC_045011.1 | Autosome |
| HiC_scaffold_34 | NC_045012.1 | Autosome |

Table S4. List of the species were used for phylogenetic analyses.

| Species | Voucher number | Sampling locality | Lending institution | Longitude | Latitude | Elevation (m) |
| --- | --- | --- | --- | --- | --- | --- |
| *Prunella atrogularis* | 141230 | Tien-Shan mts, Kyrgyzstan | NHMD | E140°51' | N39°56' | 2881 |
| *Prunella collaris* | S1318 | Taiwan | IOZ | Unknown | Unknown | Unknown |
|  | PA20150614-3 | Niubei Shan, Sichuan | IOZ | E102°53' | N29°42' | 3660 |
|  | PCM2 | Russia, Republic of Karachay-Cherkessia Kyshkadzher area | YPM | E41°42' | N43°54' | 893 |
|  | PCM749 | Russia, Krasnodarskiy Kray, Mostovskoi Rayon | UWBM | E40°36' | N44°06' | 707 |
|  | PCM765 | Russia, Krasnodarskiy Kray, Mostovskoi Rayon | UWBM | E44°36' | N44°06' | 707 |
|  | PCS2 | Greece, Crete, Chania: N foot of Mt. Trocharis | YPM | E242°01' | N35°17' | 2378 |
|  | PCS3 | Greece, Crete, Chania: N foot of Mt.Trocharis | YPM | E242°01' | N35°17' | 2378 |
| *Prunella o. fagani* | fagani | Yemen | AMNH | Unknown | Unknown | Unknown |
| *Prunella fulvescens* | QH063 | Gangcha, Qinghai | IOZ | E99°54' | N37°13' | 3196 |
|  | QHH0845 | Qinghai Lake | IOZ | E99°47' | N36°59' | 3400 |
| *Prunella himalayana* | B57883 | Dzavhan Aymag, Mongolia | UWBM | E98°53' | N47°53' | Unknown |
|  | B66660 | Respublika Tyva, Russia | UWBM | E89°87' | N50°39' | Unknown |
| *Prunella immaculate* | XZ14205 | Linzhi, Tibet |  | E94°42' | N29°39' | 3900 |
|  | sch344 | Yanbian, Sichuan | IOZ | E101°17' | N27°07' | 2200 |
|  | XZ14211 | Linzhi, Tibet | IOZ | E94°42' | N29°39' | 3900 |
| *Prunella koslowi* | 26923 | Mongolia | NHMO | Unknown | Unknown | Unknown |
| *Prunella modularis* | SK033 | Zvolen, Slovakia | IOZ | Unknown | Unknown | Unknown |
|  | SK035 | Zvolen, Slovakia | IOZ | Unknown | Unknown | Unknown |
|  | SK011 | Zvolen, Slovakia | IOZ | Unknown | Unknown | Unknown |
| *Prunella montanella* | jia42 | Maoer mountain, Heilongjiang | IOZ | E127°39' | N45°24' | Unknown |
|  | BJHR0704 | Huairou, Beijing | IOZ | E116°54' | N40°41' | Unknown |
|  | m80200 | Maoer mountain, Heilongjiang | IOZ | E127°39' | N45°24' | Unknown |
| *Prunella o. ocularis* | MR1290 | Mt. Aragats |  | E44°19' | N40°41' | Unknown |
|  | EAK479 | Mt. Aragats |  | E44°44 | N40°47' | Unknown |
| *Prunella rubeculoides* | QHH0839 | Qinghai Lake | IOZ | E99°47' | N36°59' | 3194 |
|  | QHH0835 | Qinghai Lake | IOZ | E99°47' | N36°59' | Unknown |
|  | QHH0833 | Qinghai Lake | IOZ | E99°47' | N36°59' | Unknown |
| *Prunella rubida* | B2S-63616 | Iwate Japan | YI | E140°51' | N39°56' | 1550 |
|  | 2S-63614 | Japan | YI | E140°51' | N39°56' | 1550 |
| *Prunella strophiata* | YN123 | Gaoligong, Yunnan | IOZ | E98°43' | N24°58' | 2031 |
|  | BLJ012 | Wenxian, Gansu | IOZ |  |  | 3135 |
|  | XZ15142 | Linzhi, Tibet | IOZ | E94°42' | N29°39' | 3900 |
| *Passer montanus* | SX055 | Huayin, Shannxi | IOZ | E110°06' | N34°57' | 334 |
|  | SX122 | Huayin, Shannxi | IOZ | E110°06' | N34°57' | 334 |

NHMD, Natural History Museum of Denmark, Copenhagen; NHMO, Natural History Museum, University of Oslo; AMNH, American Museum of Natural History; YPM, Yale Peabody Museum; UWBM, University of Washington Burke Museum; YI, Yamashina Institute for Ornithology.

Table S5. Resequencing information and genome wide coverage of 36 individuals used in this study.

| Lineages | Sample ID | Clean Reads Number | Proportion Above Q30(%) | Mapping Rate (%) | Sequencing Depth (×) |
| --- | --- | --- | --- | --- | --- |
| *Prunella astrogularis* | 141230 | 177392824 | 92.39 | 98.23 | 20.20 |
| *Prunella collaris* | 130562 | 134389874 | 92.16 | 91.36 | 14.43 |
|  | PA20150614-3 | 132780806 | 93.14 | 97.69 | 16.82 |
|  | PCM2 | 419239102 | 89.75 | 97.59 | 43.53 |
|  | PCM749 | 431989178 | 89.92 | 97.69 | 44.34 |
|  | PCM765 | 485605862 | 90.14 | 97.72 | 36.03 |
|  | PCS2 | 396572778 | 89.46 | 97.55 | 44.55 |
|  | PCS3 | 275614427 | 90.11 | 97.79 | 17.74 |
|  | S1318 | 141245674 | 91.46 | 97.70 | 17.21 |
| *Prunella o. fagani* | fagani | 191765016 | 85.76 | 94.85 | 14.43 |
| *Prunella fulvescens* | QH063 | 159583864 | 92.72 | 97.32 | 20.07 |
|  | QHH0845 | 155322662 | 93.41 | 97.89 | 19.01 |
| *Prunella himalayana* | B57883 | 161749920 | 91.22 | 96.40 | 19.57 |
|  | B66660 | 176874870 | 91.7 | 97.36 | 22.61 |
| *Prunella immaculate* | sch344 | 222133192 | 89.88 | 95.02 | 25.57 |
|  | XZ14205 | 235714076 | 91.18 | 95.62 | 27.90 |
|  | XZ14211 | 178485968 | 90.39 | 96.29 | 21.38 |
| *Prunella koslowi* | 26923 | 135105390 | 93.80 | 98.74 | 17.21 |
| *Prunella modularis* | SK011 | 313959874 | 90.19 | 94.80 | 33.67 |
|  | SK033 | 152470518 | 89.99 | 93.74 | 17.31 |
|  | SK035 | 131543116 | 92.04 | 98.04 | 16.63 |
| *Prunella montanella* | BJHR0704 | 173807898 | 92.11 | 97.81 | 21.53 |
|  | jia42 | 139737572 | 90.81 | 89.19 | 15.65 |
|  | m80200 | 141951232 | 94.03 | 97.50 | 12.20 |
| *Prunella o. ocularis* | EAK479 | 146171636 | 88.57 | 97.27 | 13.49 |
|  | MR1290 | 163467858 | 89.77 | 97.36 | 14.87 |
| *Prunella rubeculoides* | QHH0833 | 146085598 | 89.88 | 96.22 | 18.12 |
|  | QHH0835 | 150757516 | 91.11 | 96.52 | 18.87 |
|  | QHH0839 | 130278048 | 93.27 | 97.49 | 16.76 |
| *Prunella rubida* | 2S-63614 | 115909954 | 93.31 | 98.69 | 14.44 |
|  | B2S-63616 | 148949740 | 93.56 | 97.91 | 18.53 |
| *Prunella strophiata* | BLJ012 | 138231244 | 90.46 | 97.11 | 17.28 |
|  | XZ15142 | 125207582 | 93.75 | 98.65 | 16.25 |
|  | YN123 | 136518740 | 88.74 | 97.27 | 16.82 |
| *Passer montanus* | SX055 | 166312044 | 90.65 | 92.63 | 17.03 |
|  | SX122 | 174498706 | 92.24 | 92.71 | 18.10 |

Table S6. Gene concordance factor (gCF) for the nodes (1–7, Fig. 4a-b) that support the species tree (gCF), the two most common alternative topologies (gDF1 and gDF2), and the relative frequency of all other topologies (gDFp). The four nodes that are recovered consistently topology by the intron and exon phylogenies (*i.e.*, node 1-4) are typically supported by the majority of individual gene trees, as suggested by the high gCF values than the two gDF values. However, for the three nodes (*i.e.*, node 5-7) that fall into anomaly zone, alternative quartets (gDF1 or gDF2) occur at roughly similar to (*i.e.*, introns) or higher (*i.e.*, intron) than the major quartets (gCF).

|  | Node in species tree | gCF | gDF1 | gDF2 | gDFp |
| --- | --- | --- | --- | --- | --- |
| MP-EST (intron-set) | 1 | 0.956 | 0.009 | 0.011 | 0.024 |
|  | 2 | 0.683 | 0.081 | 0.076 | 0.161 |
|  | 3 | 0.494 | 0.099 | 0.115 | 0.292 |
|  | 4 | 0.508 | 0.009 | 0.006 | 0.478 |
|  | 5 | 0.057 | 0.041 | 0.022 | 0.880 |
|  | 6 | 0.019 | 0.011 | 0.14 | 0.955 |
|  | 7 | 0.027 | 0.017 | 0.01 | 0.947 |
| ASTRAL (intron-set) | 1 | 0.956 | 0.009 | 0.011 | 0.241 |
|  | 2 | 0.683 | 0.080 | 0.076 | 0.161 |
|  | 3 | 0.494 | 0.115 | 0.099 | 0.292 |
|  | 4 | 0.508 | 0.009 | 0.006 | 0.478 |
|  | 5 | 0.057 | 0.041 | 0.022 | 0.880 |
|  | 6 | 0.019 | 0.014 | 0.012 | 0.955 |
|  | 7 | 0.027 | 0.017 | 0.009 | 0.947 |
| MP-EST (exon-set) | 1 | 0.747 | 0.028 | 0.027 | 0.198 |
|  | 2 | 0.327 | 0.083 | 0.059 | 0.532 |
|  | 3 | 0.228 | 0.008 | 0.004 | 0.839 |
|  | 4 | 0.057 | 0.041 | 0.022 | 0.880 |
|  | 5 | 0.015 | 0.004 | 0.004 | 0.977 |
|  | 6 | 0.002 | 0.008 | 0.001 | 0.989 |
|  | 7 | 0.004 | 0.003 | 0.008 | 0.986 |
| ASTRAL (exon-set) | 1 | 0.747 | 0.028 | 0.027 | 0.198 |
|  | 2 | 0.327 | 0.083 | 0.059 | 0.532 |
|  | 3 | 0.228 | 0.044 | 0.035 | 0.693 |
|  | 4 | 0.150 | 0.008 | 0.004 | 0.839 |
|  | 5 | 0.015 | 0.004 | 0.004 | 0.977 |
|  | 6 | 0.001 | 0.008 | 0.002 | 0.989 |
|  | 7 | 0.004 | 0.008 | 0.003 | 0.985 |
